# Translating/Creating a Culturally Responsive Spanish-Language Mobile App for Visit Preparation: Case Study of “Trans-Creation”

**DOI:** 10.2196/12457

**Published:** 2019-04-05

**Authors:** Denise Ruvalcaba, Hidemi Nagao Peck, Courtney Lyles, Connie S Uratsu, Patricia R Escobar, Richard W Grant

**Affiliations:** 1 Division of Research Kaiser Permanente Northern California Oakland, CA United States; 2 Regional Health Education The Permanente Medical Group Oakland, CA United States; 3 Division of General Internal Medicine Zuckerberg San Francisco General Hospital and Trauma Center University of California San Francisco, CA United States; 4 Center for Vulnerable Populations University of California San Francisco, CA United States

**Keywords:** IT tool development, transcreation, doctor-patient communication, primary care

## Abstract

**Background:**

Health information technology (IT) tools are increasingly used to improve patient care. However, implementation of English-only health IT tools could potentially worsen health disparities for non-English speakers.

**Objective:**

We aim to describe the “trans-creation” process of developing linguistically and culturally appropriate health IT tools through a detailed case analysis of a waiting room health mobile app designed to help Spanish-speaking Latino people prepare for primary care visits.

**Methods:**

We adapted the English-language Visit Planner mobile app for Spanish-speaking Latino patients. We applied culturally defined themes derived from prior published research and input by both skilled linguists and potential end users. Initial changes were iteratively reviewed and edited by a team of writers, health care educators, subject matter experts, patients, and providers.

**Results:**

The trans-creation process resulted in the following key culturally mediated changes to the tool: replacing the “provider” actors with “patient” actors; changing the choice of “Stress at Home or Work” (represented by an icon of a house) to “Mi Familia” (translation: my family; icon is an outline of family members holding hands); replacing the English terms “anxiety” and “depression” with “Me siento desanimado”(translation: I am feeling down) to avoid mental health stigma; and using more concise text translation to ensure the wording fit the available on-screen space.

**Conclusions:**

The trans-creation process of cultural and linguistic adaptation led to several design changes that would not have been implemented if we had simply translated the words from English to Spanish.

## Introduction

Latino people are the fastest and largest growing minority group in the United States and are projected to account for one-third of the US population by 2060 [1]. Latino people face high rates of chronic conditions like heart disease, diabetes, and cancer [2]. In 2010, the Centers for Disease Control and Prevention reported that 14.2% of Latinos had two or more concurrent chronic conditions [3]. Given this high prevalence of chronic illness and the projected growth of the Latino population, increased efforts are needed to address how to improve health outcomes for this community.

Effective patient-provider communication is the cornerstone of good health care [4,5]. Patients with diabetes who report good provider-patient communication, for example, have been shown to have better self-care and glycemic control [6,7]. Language barriers represent an important source of health disparities among Latino people [8]. Indeed, communication between English-speaking doctors and Latino patients may often be hampered because 30%-40% of the Latino population speaks English less than “very well” (often defined as limited English proficiency) [9]. Beyond simple language discrepancy, culture also plays a key role in the health of Latino people, because there are culture-specific understandings of illness including stigma associated with certain conditions such as mental health [10]. Research has identified several core cultural constructs such as *f* *amilismo* (the central role of family) and *confianza* (importance placed on trusting health care providers) that are particularly relevant to health-related interventions [11,12]. Effective communication with Latino patients must therefore also consider the cultural framework within which Latino people experience their health care.

Health information technology (IT) tools are increasingly being used within health care settings and have been proven to enhance patient-provider communication and lead to better health outcomes [13,14]. However, English-only health IT tools could potentially increase health disparities if they only benefit English speakers. Therefore, developing culturally responsive Spanish-language health IT tools is imperative. As part of a funded project (PCORI/CDR-1403-11992), we previously developed an English-language health mobile app (Visit Planner) to help patients prepare for their primary care visits while in the waiting room [15]. To adapt our tool for Spanish-speaking Latino people, we applied the process of “trans-creation” (derived from combining “translation” and “creation”) to culturally and linguistically adapt materials for the target population [16]. Here, we describe the process, changes, and impact of this trans-creation process.

## Methods

### Setting

This study was conducted within Kaiser Permanente Northern California (KPNC), a nonprofit integrated-care delivery system providing care for over 4.1 million members throughout Northern California. KPNC serves individuals from diverse demographic and socioeconomic backgrounds, including over 700,000 members who self-identify as Latino. The Kaiser Foundation Research Institute Institutional Review Board approved the study.

### Visit Planner - English Version

The Visit Planner was first created in English for the “Aligning Patients and Providers” study (trial registration: ClinicalTrials.gov NCT02707146), which is described in detail elsewhere [15]. Briefly, we applied user-centered design principles such as stakeholder involvement and iterative modifications using wire diagrams and paper versions to incorporate end-user feedback in order to develop an iPad waiting room tool (Visit Planner). This tool supported patients in identifying and discussing their top priorities for their primary care visit. Using the Flesch-Kincaid Grade-Level tests, the reading level of the tool was set between a 4th to 6th grade level to ensure accessibility for a broad audience.

The Visit Planner begins with a 30-second video that emphasizes the importance of identifying and discussing top health concerns early in the visit. The next screen prompts the patient to select his/her top two areas of concern out of the six available options (New Problem, Old Problem, Medicines, Stress at Home or at Work, A Personal Concern or Other, Need Something From my Doctor). Once the patient selects one or two option(s), three to four suboptions are offered under each selection. For example, if the patient selects “Medicines” as one of the concerns, three options are presented: (1) Problems With Side Effects, (2) Medicines Cost Too Much, and (3) Stopped Taking. After the patient decides on his/her top priorities for the visit, a second brief video advises the patient to take notes and ask questions during the visit. The actress in both English-language videos portrays a health care provider. The final screen of the Visit Planner is a summary of the patient’s selected visit priorities and strategies, which is printed out and given to the patient to help with the ensuing visit. ** **

### Trans-Creation: A Step-by-Step Process

**Figure 1:**
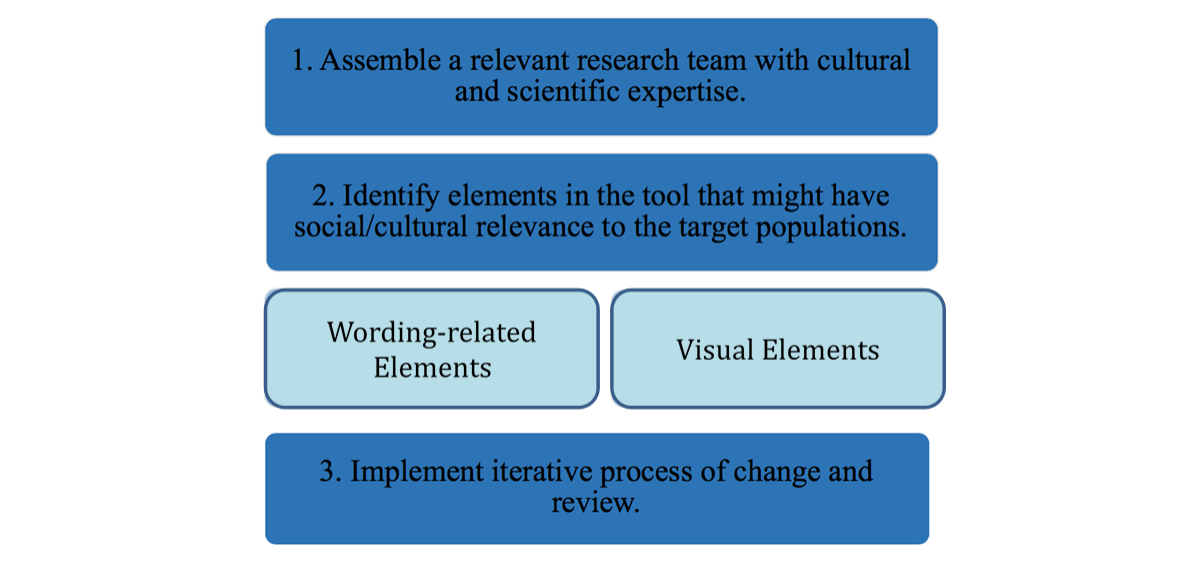
The three-step transcreation model.

To better serve the growing Latino population, our research team partnered with health educators and Latino cultural brokers to adapt the Visit Planner for Spanish-speaking Latino people by using the following three-step trans-creation model (Figure 1).

#### Step 1: Assemble the Team

Team members were chosen based on the concept of “Constituent-Involving,” which is the process of hiring and training staff members from within the target population [16]. These cultural brokers provided important insight into nuanced cultural knowledge gained from personal experience. Key team members were hired after they passed a Spanish-language fluency test, a step not usually practiced in other trans-creation/translation projects, but which we found vital for creating an authentic product. Five individuals with relevant skills for this project (three Latino Spanish-language editors, one Latina bilingual health education specialist, and one medical anthropologist) were added to the original research team. The Spanish-language editors ensured that the intent, tone, content strategy, and linguistic fidelity of the Spanish version had as high quality as the original version; the education specialist provided important input on effective communication strategies to reach the target audience; and the medical anthropologist provided cross-discipline expertise in cultural responsiveness and public health interventions.

#### Step 2: Identify Relevant Language and Visual Elements

Our team of cultural experts analyzed the original “Pre-Visit Tool” with a focus on wording, visual elements, and culturally sensitive content area. This process included using the tool itself as well as reviewing transcripts of the video segments within the tool. The editorial staff was specifically trained to analyze the health literacy levels of the general population and to tailor the text to ensure its accessibility to most patients. Analysis of visual elements included assessment of icons, color, and video presentation. Finally, the team identified conceptual areas within the tool where Latino cultural values might influence how the Spanish version of the tool should be created.

#### Step 3: Testing and Iteration

After the initial process of translation and the changes were made based on visual and cultural elements, the Spanish content was back translated into English to ensure that none of the original intended meaning was lost. Scripts for the video clips were also revised in real-time during filming to ensure optimal comprehension.

### Assessing Use of the Mobile App

The Spanish version of the Visit Planner was implemented at two practices within KPNC as part of the larger, externally funded “Aligning Patients and Providers” clinical trial. Time patients spent using the tool was recorded, and a postvisit survey about the tool was administered that asked patients about their visit experiences after using the Pre-Visit Planner. Survey questions reported here were adapted from the Stanford Communication with Physicians Scale [17]. Comparison of results between English- and Spanish-language tool users was made using *t* test or Chi-square tests, as appropriate.

## Results

Our trans-creation process resulted in several key changes related to both the wording of the text and the visual representation on the screen.

### Wording Modifications

#### Family

In Latino culture, family (both immediate and extended family members) is often highly valued and can play a key role in medical care [18]. The importance of family as a cultural influence led our research team to embed concepts of family throughout the Visit Planner tool. For example, the team changed the visit priority option “Stress at Home or Work” to “Mi Familia” (translation: my family) to acknowledge the emphasis Latino culture places on family and multigenerational homes. Another change to emphasize the role of family was to replace “caregiving issues” as a concern to the more specific “No sé cómo atender a un ser querido” (translation: I don’t know how to care for a loved one).

#### Literacy

The English version of the Visit Planner was designed to have a 4th to 6th grade reading level using the Flesch-Kincaid grade-level assessment tool. Because there is currently no validated tool to assess Spanish reading grade level, we formally assessed the literacy level by translating our final Spanish text back into English and then applying the Flesch-Kincaid grade-level assessment. Health-specific literacy was also an important consideration. For example, we changed the phrase “Referral to specialist” to “Que me diga si necesito un especialista” (translation: Tell me if I need a specialist) to remove the word “referral” due to the concern that patients with less medical literacy may not immediately understand the term.

#### Stigma

During the trans-creation process, the team is trained to consider best practices for a given cultural community. This knowledge included identifying that mental health can be a particularly difficult subject to discuss. Indeed, prior research has shown that Latino people are less likely to inquire about and receive mental health care due to the stigma associated with mental health [19,20]. Accordingly, under the topic of “Personal Concerns,” the choices of “anxiety” and “depression” in the English version were changed to the less stigma-associated “Me siento desanimado” (translation: I am feeling down). Use of this euphemism was designed to encourage Latino patients to talk about emotional health and help those who may not recognize that certain symptoms they experience may be related to anxiety or depression.

#### Concise Text

Spanish tends to require more syllables to convey the same direct wording as English [21]. Thus, in several cases, we needed to change the wording of the original English text before translating it to Spanish. For example, one of the choices in the “Need something more from my doctor” category was “Blood test, x-ray, or other test” (32 spaces). Direct translation of these words into Spanish yields “Prueba de sangre, rayos X u otra prueba” (39 spaces, 18% longer). In this case, we adapted the English version to “Ask if I need more tests,” which translates to Spanish as “Confirmar si necesito mas pruebas” (33 spaces).

### Visual Modifications

#### Actors

**Figure 2:**
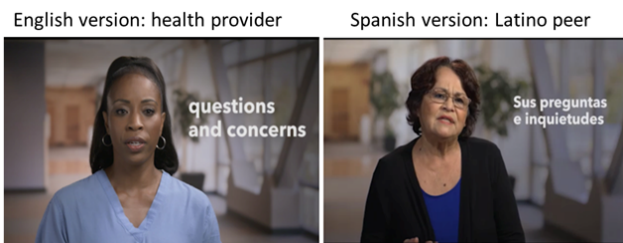
Actors in the Spanish version were switched with a peer.

In the original Visit Planner, the actors in the video clips portrayed a “health provider,” whereas in the videos for the Spanish-language version, the actors portrayed fellow patients (Figure 2). In conjunction with this patient-level portrayal, the script was modified, so that the coaching advice was framed as “en mi experiencia” (translation: In my experience), because prior research has shown that peer-to-peer advising is more effective within the Latino population [22]. The change in phrasing was also intended to increase *c* *onfianza* (trust). The actors were selected to resemble the typical age and appearance of our Latino patients, and the actors were instructed to dress casually and choose everyday wear for the video clips.

#### Icons

**Figure 3:**
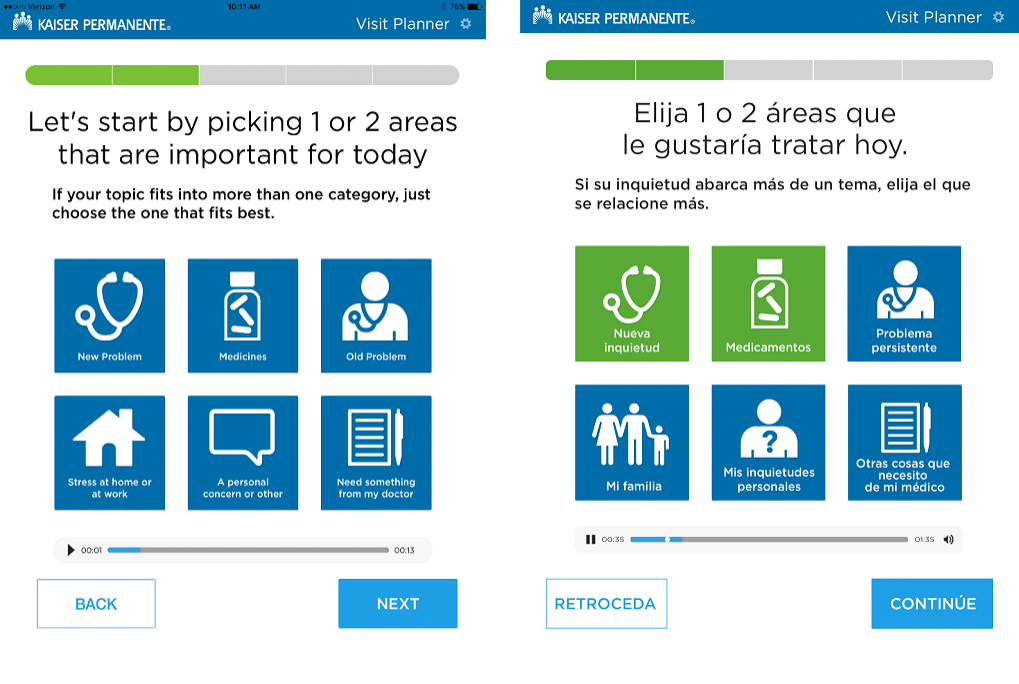
Screenshots of the English and Spanish versions of the primary prioritization page.

All the icons from the English Visit Planner were used in the Spanish version, with the exception of the icon originally associated with “Stress at home or work,” which in the English version, was an outline of a house. To better align with the changed text (the English equivalent of “my family”), we also changed the icon to an outline of a family (Figure 3).

### Comparison of the Spanish and English Visit Planner Versions

Spanish-speaking patients (n=41) spent a similar amount of time going through the Spanish version of the Visit Planner as users of the original English tool (n=273; 5.26 minutes vs 5.05 minutes; *P*=.48). In the postvisit survey, responses were similar between groups (Table 1), with the exception that Spanish speakers were more likely to discuss their visit experience with friends and loved ones than English speakers (35/41 [86%] vs 188/273 [69%], *P*=.02).

**Table 1:** Postvisit survey results comparing patients using the English version and those using the Spanish version of the Visit Planner Tool.

Questions	Proportion of respondents answering “Yes”		*P* value
	English version (N=273), n (%)	Spanish version (N=41), n (%)	
Did you ask questions about the things you want to know and the things you don’t understand about your treatment?	259 (95)	36 (88)	.09
Did you tell your doctor about your top concerns at the beginning of the visit?	257 (94)	37 (91)	.39
Did you take notes or ask questions during your visit?	246 (90)	33 (81)	.11
After a visit, did you talk to friends or loved ones about the visit?	188 (69)	35 (86)	.02
Did you discuss any personal problems that may be related to your illness?	175 (64)	26 (63)	.89

## Discussion

In this case study, we describe the process of adapting an English-language health mobile app for Spanish-speaking Latino people. Because merely translating health education materials from one language to another does not address cultural differences in how medical care is perceived by patients from different backgrounds, we implemented a three-step trans-creation process to identify, reinforce, and build upon the Latino cultural values and concepts [23]. Trans-creation steps included (1) adding members to the research team with appropriate linguistic and cultural expertise, (2) carefully assessing every element of the tool for its potential cultural significance, and (3) iteratively testing and assessing the resulting changes. This iterative process required five distinct rounds of testing and associated modifications.

Efforts to design effective health IT tools are highly relevant to addressing the problem of health disparities within the large and growing US Latino population. More than 50 million Latinos in the United States have limited English proficiency and face the possibility of being left further behind as new health IT tools are developed to improve doctor-patient communication and other aspects of health care [9]. Our Visit Planner study represents one of the first published examples of applying the trans-creation process to a waiting room, iPad-based health mobile app designed to help patients more effectively communicate with their providers during visits. The lessons learned in this process, while specific to our Visit Planner tool, provide illustrative examples of the broad issues that may be faced by others planning to create educational tools for Latino people.

Many of the changes we made were reflective of several core cultural constructs that have been previously described in the US Latino population. These constructs, applied to the Visit Planner trans-creation process, included *familismo* (family), *confianza* (trust), and the role of mental health stigma. The key changes that we made in our Visit Planner (eg, altering language and images to emphasize family, replacing “medical” actors with “peer” actors to increase trust and softening the language used to describe anxiety and depression to reduce stigma), all exemplify how the trans-creation process adds crucial enhancements beyond mere translation. Lessons we learned during this process are all potentially transferable to other trans-creation projects.

Initial evaluation of how patients used the Visit Planner tool revealed that Latino users of the Spanish version spent a similar amount of time as they did on the English version, to work through the sequential screens, indicating that we were successful in keeping the time and effort required to complete the Visit Planner similar in the two versions. Moreover, the survey responses completed after the visit indicated that both English- and Spanish-speaking users had similar experiences during the visit. The only difference between groups was the greater discussion with family among Latino people, which provides further empiric evidence to support our emphasis on *familismo.*

In the English-language version, our team asked participants about mental health symptoms directly; however, this method was not appropriate for the Spanish-language version. In Latino culture, mental health problems are not necessarily validated, are often seen as a sign of weakness, and may carry stigma [20]. In fact, although the rates of mental illness are similar among white and Latino people, the latter are much less likely to seek health care for mental health concerns [20]. Association with a mental illness may even reflect poorly on the family and place stigma not only on the individual but also their relatives [22]. Concern about this stigma influenced our trans-creation process in a way that would not have occurred with simple translation.

A recent review of health IT implementation for Latino people demonstrated several important gaps in the literature, which our study helps to fill [24]. This review found that only 60% of studies targeted toward Spanish-speaking Latino people were culturally tailored. Increasing culturally tailored health IT is necessary because a culture-specific platform can better engage patients and improve health outcomes. This narrative synthesis also highlighted the crucial need for future studies to explicitly detail their cultural tailoring process and support it with evidence-based literature. Our study does this by providing a model to follow and a description of our process; future studies can more easily replicate cultural tailoring with our trans-creation model and description.

Two limitations of our study deserve mention. First, because of the funding sequence, the English version was created first and used as a template for the Spanish version. Many experts recommend that culturally tailored materials be created *de novo* or in tandem, rather than as variations on existing materials [25,26]. Second, the results of our randomized clinical trial are not yet available; therefore, we cannot report on the clinical impact of our tool.

Beyond simple translation, trans-creation provides a powerful framework for adapting English health IT tools for patients from different cultures who speak different languages. Our trans-creation process, while specific to the Visit Planner tool, demonstrated several key principles that can be applied to further the reach of health education tools for patients from non-English speaking cultures.

## Abbreviations

IT: information technology
KPNC: Kaiser Permanente Northern California
